# Activation of an anti-bacterial toxin by the biosynthetic enzyme CysK: mechanism of binding, interaction specificity and competition with cysteine synthase

**DOI:** 10.1038/s41598-017-09022-6

**Published:** 2017-08-18

**Authors:** Roberto Benoni, Christina M. Beck, Fernando Garza-Sánchez, Stefano Bettati, Andrea Mozzarelli, Christopher S. Hayes, Barbara Campanini

**Affiliations:** 1 Dipartimento di Medicina e Chirurgia, Università di Parma, Parma, Italy; 2 Department of Molecular, Cellular and Developmental Biology, University of California, Santa Barbara, Santa Barbara, CA USA; 3 Istituto Nazionale Biostrutture e Biosistemi, Rome, Italy; 4 Dipartimento di Scienze degli Alimenti e del Farmaco, Università di Parma, Parma, Italy; 5 Biomolecular Science and Engineering Program, University of California, Santa Barbara, Santa Barbara, CA USA; 6 Institute of Organic Chemistry and Biochemistry of the Czech Academy of Sciences, Praha, Czech Republic; 7 Icahn School of Medicine at Mount Sinai, New York, NY USA

## Abstract

Contact-dependent growth inhibition (CDI) is a wide-spread mechanism of inter-bacterial competition. CDI^+^ bacteria deliver CdiA-CT toxins into neighboring bacteria and produce specific immunity proteins that protect against self-intoxication. The CdiA-CT toxin from uropathogenic *Escherichia coli* 536 is a latent tRNase that is only active when bound to the cysteine biosynthetic enzyme CysK. Remarkably, the CysK:CdiA-CT binding interaction mimics the ‘cysteine synthase’ complex of CysK:CysE. The C-terminal tails of CysE and CdiA-CT each insert into the CysK active-site cleft to anchor the respective complexes. The dissociation constant for CysK:CdiA-CT (*K*_d_ ~ 11 nM) is comparable to that of the *E. coli* cysteine synthase complex (*K*_d_ ~ 6 nM), and both complexes bind through a two-step mechanism with a slow isomerization phase after the initial encounter. However, the second-order rate constant for CysK:CdiA-CT binding is two orders of magnitude slower than that of the cysteine synthase complex, suggesting that CysE should outcompete the toxin for CysK occupancy. However, we find that CdiA-CT can effectively displace CysE from pre-formed cysteine synthase complexes, enabling toxin activation even in the presence of excess competing CysE. This adventitious binding, coupled with the very slow rate of CysK:CdiA-CT dissociation, ensures robust nuclease activity in target bacteria.

## Introduction

Though long considered to be isolated and independent unicellular organisms, bacteria engage in a multitude of cooperative and competitive behaviors. Many bacteria secrete soluble antibiotics and bacteriocins^1–4^, which diffuse through the environment and kill competing bacteria at a distance. More recently, proximity-dependent inter-bacterial competition systems have been characterized^5–8^. This phenomenon was first described in *Escherichia coli* isolate EC93, which inhibits the growth of other *E. coli* strains in a contact-dependent manner^5^. Contact-dependent growth inhibition (CDI) is mediated by CdiB/CdiA two-partner secretion proteins, which transfer protein toxins between Gram-negative bacteria^9, 10^. CdiB is an outer-membrane transport protein that exports CdiA onto the cell surface. CdiA forms a long β-helical filament that extends from the inhibitor cell to bind specific receptors on neighboring bacteria. Upon binding receptor, CdiA delivers its C-terminal toxin domain (CdiA-CT) into the target cell to inhibit growth. CDI^+^ bacteria also express CdiI immunity proteins, which bind to the CdiA-CT domain and neutralize toxin activity to prevent self-intoxication. Analysis of CdiA from many species has revealed that the family carries a wide variety of C-terminal toxin domains, each with a distinct activity^11–15^. Thus, a given CdiI immunity protein only protects against its cognate toxin and not the toxins deployed by other bacteria. Together, these observations suggest that CDI systems mediate inter-bacterial competition for growth niches and other environmental resources.

We recently discovered that the CDI toxin deployed by uropathogenic *E. coli* 536 is a latent tRNase that is only active when bound to the biosynthetic enzyme CysK^16^. CysK is a pyridoxal 5′-phosphate (PLP)-dependent *O*-acetyl-L-Ser sulfhydrylase that catalyzes the last step of cysteine biosynthesis in eubacteria, plants and some archaea^17–19^. *E. coli* and many other bacteria encode an additional isozyme, CysM. Both sulfhydrylases are coordinately regulated with the enzymes responsible for sulfate reduction to bisulfide^17^, although the functional role of CysM is less well characterized. The structure and catalytic properties of CysK from Gram-negative bacteria and plants have been characterized thoroughly^20–33^. CysK has long been known to form a high-affinity “cysteine synthase” (CS) complex with CysE, which is a serine *O*-acetyltransferase responsible for the penultimate reaction in cysteine biosynthesis^34–37^. The three-dimensional structure of the cysteine synthase complex is unknown, but biochemical studies indicate that each CysE hexamer binds to two CysK homodimers^38^. Moreover, it is well established that the flexible C-terminal tail of CysE inserts into the CysK active site to anchor the complex^36, 37, 39^. The C-terminal Ile residue of CysE is particularly critical, and deletion or mutation of this conserved residue consistently interferes with cysteine synthase assembly^39, 40^. The Ile side-chain mimics substrate to bind the CysK active site, and consequently the cysteine synthase complex is dissociated with micromolar concentrations of *O*-acetyl-L-Ser^34, 41^. Remarkably, the *E. coli* 536 CdiA-CT toxin mimics CysE and uses its C-terminal peptide motif to bind the CysK active site^16, 42^. In fact, a number of proteins engage in so-called “moonlighting” binding interactions with CysK homologs^43^. One intriguing example is the EGL-9 prolyl hydroxylase from *Caenorhabditis elegans*, which uses its C-terminal Ile residue to interact with CYSL-1, a CysK homolog that has lost biosynthetic activity but retains the bisulfide-binding site. Under hypoxic conditions, bisulfide accumulates and promotes EGL-9:CYSL-1 binding. The sequestered EGL-9 is no longer able to hydroxylate HIF-1, which stabilizes the transcription factor and increases the expression of genes required to respond to hypoxia^44^. Thus, CysK homologs have been co-opted to regulate diverse biological activities.

Here, we explore the thermodynamics and kinetics of the CdiA-CT interaction with *E. coli* CysK (EcCysK) to gain insight into toxin activation. We find that the dissociation constant for the EcCysK:CdiA-CT complex is comparable to that of the CS complex, suggesting that EcCysE could attenuate toxicity by competing with CdiA-CT for access to EcCysK. In addition, the second-order rate constant for cysteine synthase complex formation is ~200-fold greater than the rate constant for EcCysK:CdiA-CT binding. Though cysteine synthase assembly is kinetically favored, CdiA-CT toxin is still activated in the presence of competing EcCysE. Robust toxin activation reflects the ability of CdiA-CT to displace EcCysE from pre-formed cysteine synthase complexes. This property, coupled with the very slow rate of EcCysK:CdiA-CT dissociation, ensures toxin activation upon entry into target bacteria. Finally, we show that CysK homologs from different bacterial species support CdiA-CT toxin activity to varying degrees. Although CDI-mediated toxin delivery only occurs between closely related bacteria^45, 46^, CdiA-CT toxin homologs are found in several species and therefore must interact with different CysK variants. Because CDI genes are acquired through horizontal gene transfer^47, 48^, we propose that the toxin domain evolved to bind a highly conserved partner that is ubiquitous in bacteria.

## Results

### Two CdiA-CT toxin domains bind each CysK dimer

**Figure 1:**
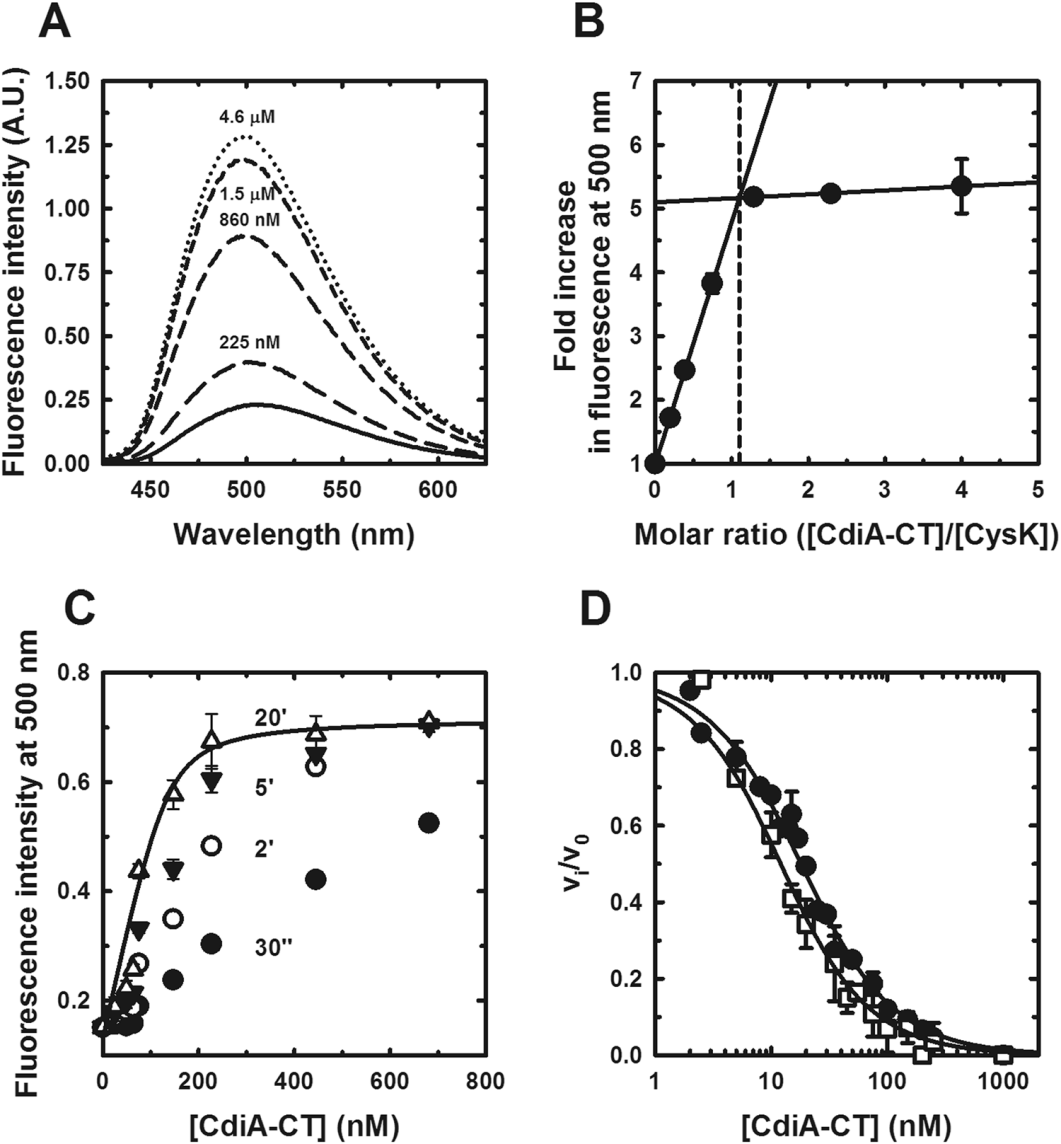
EcCysK:CdiA-CT complex formation. (**A**) Fluorescence emission spectra of EcCysK excited at 412 nm. Spectra were collected with EcCysK (1.15 µM) in the presence of the indicated concentrations of CdiA-CT. (**B**) Stoichiometry of the EcCysK:CdiA-CT complex. 1 μM EcCysK was titrated with CdiA-CT to saturation. The intersection of the lines corresponds to a CdiA-CT:EcCysK ratio of 1.1:1 (dashed line). (**C**) Determination of the EcCysK:CdiA-CT dissociation constant. EcCysK (80 nM) was titrated with increasing concentrations of CdiA-CT, and fluorescence emission at 500 nm monitored at the indicated times. The solid line indicates the Eq. 2 fit to the 20 min data set, with *K*_d_ = 10 ± 11 nM and [CysK] = 134 ± 33 nM. (**D**) CdiA-CT inhibits EcCysK sulfhydrylase activity. EcCysK (6 nM) was titrated with CdiA-CT and sulfhydrylase activity measured as described in the Methods. Eq. 3 was fitted to the dependence of v_i_/v_0_ on CdiA-CT concentration, yielding an apparent IC_50_ that was used to calculate a *K*_i_ of 11.0 ± 0.4 nM (closed circles). A *K*_i_ of 6.4 ± 0.5 nM was calculated under the same conditions in the presence of 2.4 µM CdiI (open squares).

Our previous work has shown that the CdiA-CT toxin domain forms a stable complex with EcCysK and that this interaction is required for toxic tRNase activity *in vivo* and *in vitro* ^16^. The initial study suggested that the C-terminal tail of CdiA-CT inserts into the EcCysK active site, and this conclusion was recently confirmed by crystal structures of the EcCysK:CdiA-CT complex^42^. Therefore, we reasoned that EcCysK:CdiA-CT complex formation could be monitored by measuring changes in pyridoxal 5′-phosphate (PLP) fluorescence. This spectroscopic approach has been used extensively to track cysteine synthase complex formation^38^ as well as to identify CysK inhibitors^49–54^. Indeed, PLP fluorescence increased about five-fold when EcCysK was titrated with increasing concentrations of CdiA-CT (Fig. 1A). This effect is very similar to that observed for the cysteine synthase complexes of *Haemophilus influenzae* ^38^ and *E. coli* ^55^. Moreover, the fluorescence spectrum of EcCysK:CdiA-CT exhibited a blue-shift in the emission maximum from 505 to 498 nm compared to free EcCysK (Fig. 1A). This latter change indicates that the fluorophore is in a less polar environment, consistent with insertion of the toxin’s C-terminal Ile residue into the EcCysK active site. Stoichiometric titrations determined a molar ratio of 1.1:1 (Fig. 1B), in agreement with crystal structures showing two CdiA-CT domains bound to each EcCysK dimer^42^.

### CdiA-CT and CysE bind to CysK with comparable affinity

The affinity of the EcCysK:CdiA-CT complex can be estimated through titrations of dilute EcCysK with toxin. We observed the same spectroscopic changes upon CS complex formation^56^, but the emission spectra evolved over time, stabilizing after about 20 min at the lowest toxin concentrations (Fig. 1C). Fitting of Eq. 2 to binding data collected after 20 min yields an estimated *K*_d_ of about 10 nM (Fig. 1C). The protein concentrations required for fluorescence-based titrations limit the measurable dissociation constants to about 5 nM. Therefore, we used an orthologous assay to measure binding interactions more accurately. Because CdiA-CT occludes the EcCysK active site, complex formation can be monitored by measuring fractional sulfhydrylase activity as a function of toxin concentration (Fig. 1D). The half maximal inhibitory concentration (IC_50_) of 15.4 ± 0.6 nM was obtained by fitting Eq. 3 to these data. Accounting for substrate concentration and the *K*_M_, the IC_50_ was converted to an inhibition constant (*K*_i_) of 11.0 ± 0.4 nM using Eq. 4. This value is in agreement with the EcCysK:CdiA-CT binding constant calculated from surface plasmon resonance data^57^. Moreover, EcCysE inhibits EcCysK activity with a *K*_i_ of 6.2 ± 0.7 nM^55^, indicating that the toxin and EcCysE bind to EcCysK with similar affinities.

CdiI immunity protein binds specifically to CdiA-CT toxin, but also forms a ternary complex with CdiA-CT and EcCysK^16, 42^. A recent report has suggested that the CdiA-CT:CdiI complex has a six-fold higher affinity for EcCysK than CdiA-CT toxin alone^57^. However, the crystal structure of the EcCysK:CdiA-CT:CdiI ternary complex shows that the immunity protein makes no direct contacts with EcCysK^42^. To monitor the influence of CdiI on EcCysK:CdiA-CT complex formation, we measured EcCysK sulfhydrylase activity in the presence of CdiA-CT and excess CdiI (Fig. 1D). The calculated *K*_i_ in the presence of CdiA-CT and CdiI was 6.4 ± 0.5 nM, indicating a similar affinity for EcCysK under these conditions.

**Figure 2:**
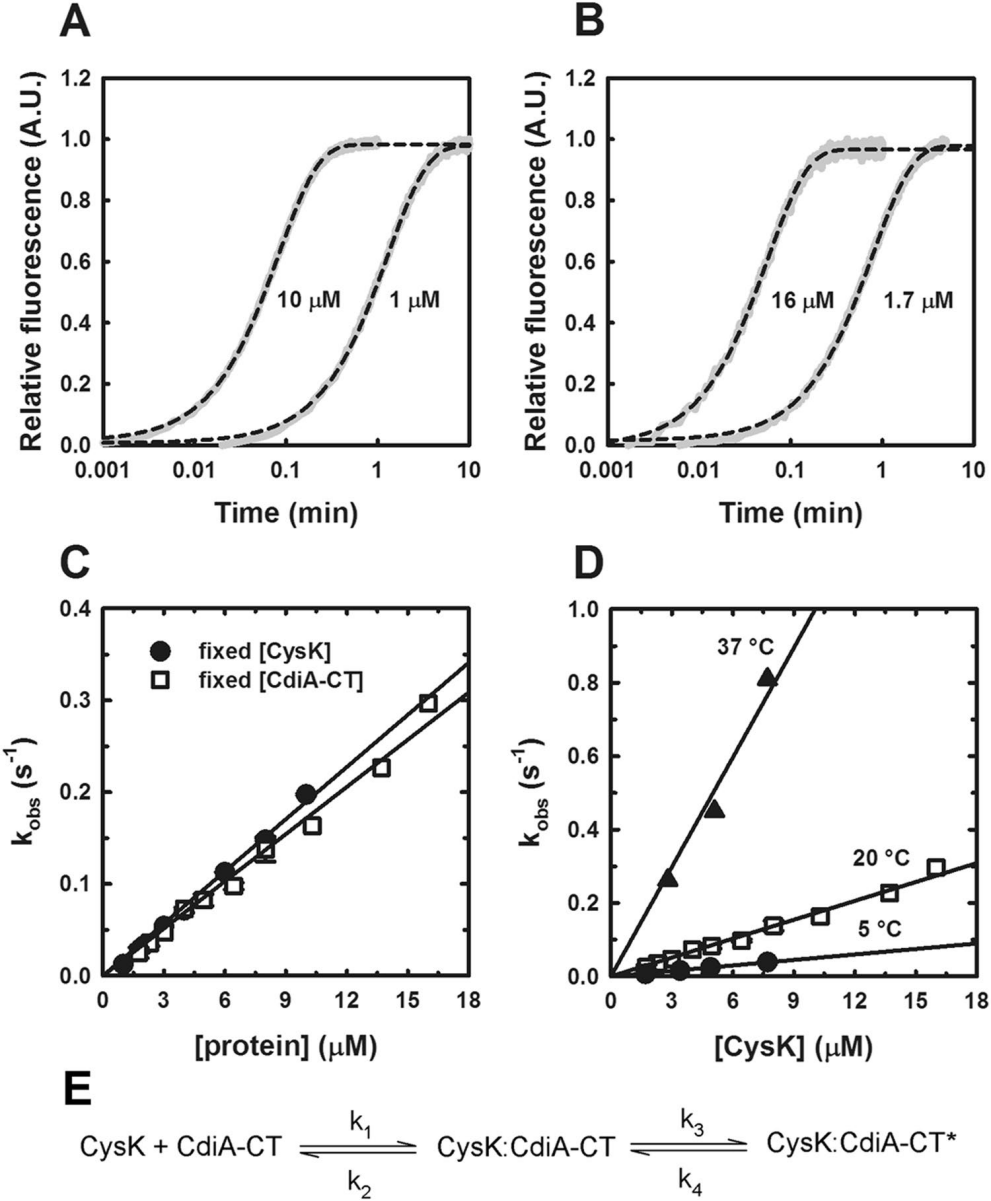
Pre-steady state kinetics of EcCysK:CdiA-CT assembly. (**A**,**B**) Representative time courses for the interaction of CdiA-CT (1 µM and 10 µM) with EcCysK (200 nM) (panel A) and EcCysK (1.7 µM and 16 µM) with CdiA-CT (270 nM) (panel B) as monitored by fluorescence emission intensity upon excitation at 412 nm. Individual traces are presented in grey, and the dashed black lines represent Eq. 5 fits to the time-course binding data. (**C**) Dependence of the observed kinetic constant (k_obs_) on EcCysK and CdiA-CT concentrations. The lines represent linear equation fits with slopes of 0.019 ± 0.007 µM^−1^·s^−1^ and 0.017 ± 0.007 µM^−1^·s^−1^. (**D**) Observed kinetic constant as a function of CysK concentration and temperature. Solid lines represent linear equation fits with slopes of 0.099 ± 0.019 µM^−1^·s^−1^ (37 °C), 0.017 ± 0.007 µM^−1^·s^−1^ (20 °C) and 0.005 ± 0.0003 µM^−1^·s^−1^ (5 °C). (**E**) Two-step model for EcCysK:CdiA-CT complex formation including a slow conformational change. CysK:CdiA-CT is the encounter complex, and CysK:CdiA-CT* corresponds to the isomerized, nucleolytic complex.

The slow binding of EcCysK and CdiA-CT prompted an examination of complex formation under pre-steady state conditions to calculate microscopic rate constants^56, 58^. We used stopped-flow spectroscopy to measure PLP fluorescence emission as a probe of CdiA-CT:EcCysK complex formation. Under the buffer and temperature conditions used for equilibrium binding, we varied EcCysK or CdiA-CT concentrations under pseudo-first order conditions, keeping the concentration of the binding partner constant (Fig. 2A,B). A single exponential equation (Eq. 5) was sufficient to fit all the kinetic traces. The k_obs_ values calculated from Eq. 5 show a linear dependence on protein concentration with slopes of about 0.02 µM^−1^·s^−1^ (Fig. 2C). This linear relationship may indicate a single-step binding mechanism, but could also be obtained from a two-step process under conditions that do not allow saturation of the effect^59^. Because the dependence was linear independent of temperature between 5 °C and 37 °C (Fig. 2D), we were unable to ascertain whether EcCysK:CdiA-CT complex formation is limited by a conformational change. However, given the unusually slow binding kinetics, rapid formation of an encounter complex followed by a slow conformational rearrangement is likely (Fig. 2E). As previously observed for the cysteine synthase (CS) complex^56^ the initial fast step was not associated with changes in the fluorescence emission, and therefore only the slow rate-limiting process can be measured by this technique. According to this model, the dependence of k_obs_ on protein concentration represents the linear portion of the hyperbole and is equal to the second-order rate constant (k_3_/*K*_d_). Fitting Eq. 6 to the dependence yields k_3_/*K*_d_ of 2·10^4^ M^−1^·s^−1^. By contrast, the second-order rate constant for the *H. influenzae* cysteine synthase complex is about 10^7^ M^−1^·s^−1^ ^56^ and 4·10^6^ M^−1^·s^−1^ for the EcCysE:EcCysK complex (Fig. S1). Thus, the EcCysK:CdiA-CT complex forms ~200-fold more slowly than the *E. coli* cysteine synthase complex.

### CdiA-CT competes with CysE for binding to CysK

**Figure 3:**
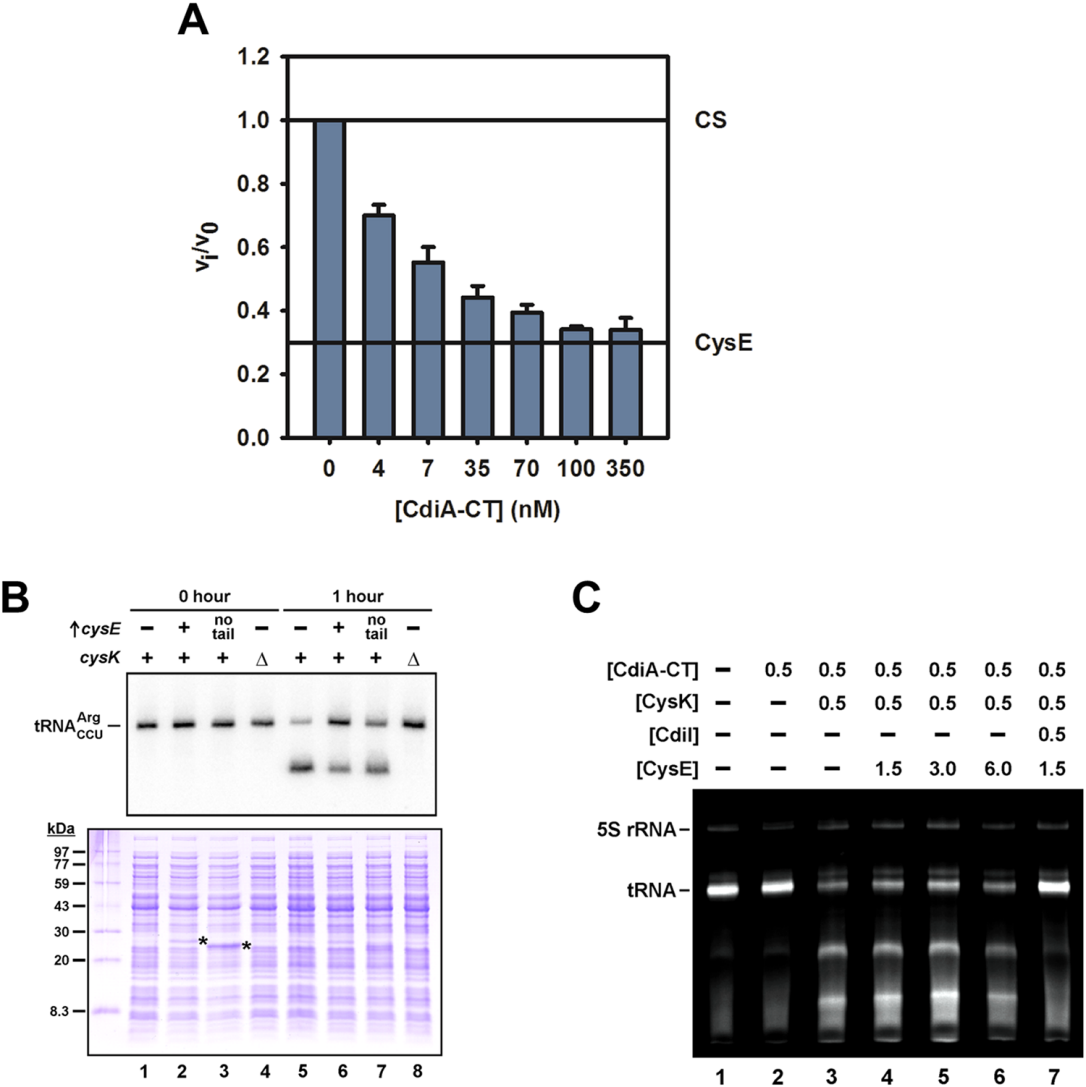
EcCysE and CdiA-CT compete for binding to CysK. (**A**) CdiA-CT blocks formation of the cysteine synthase complex. Increasing concentrations of CdiA-CT were pre-incubated with EcCysK (19 nM) for 20 min prior to the addition of L-Ser (20 mM) and EcCysE (28 nM). Reactions were then initiated by addition of 0.3 mM acetyl-CoA and acetyltransferase activity measured by monitoring the decrease in acetyl-CoA absorption at 232 nm as described in the Methods. The activities of isolated CysE and CysE in the CS complex are shown by horizontal reference lines. (**B**) EcCysE inhibits CdiA-CT toxin activation *in vivo*. Inhibitor and target cells were co-cultured as described in the Methods. Total RNA and protein were isolated upon initial mixing and after 1 h, and analyzed by Northern blot (top panel) and SDS-PAGE (bottom panel). Where indicated, target cells over-produced EcCysE (**+**) or truncated EcCysE lacking the C-terminal tail (no tail). Target cells carried a deletion of the *cysK* gene (**∆**) where indicated. Asterisks (*) in the bottom panel indicate over-produced EcCysE proteins. (**C**) EcCysE inhibits CdiA-CT toxin activation *in vitro*. EcCysK and EcCysE were pre-incubated at the indicated concentrations (µM) prior to addition of CdiA-CT and RNA substrate as described in the Methods. Reactions were quenched after 10 min at 37 °C, then run on 8 M urea-polyacrylamide gels and visualized by ethidium bromide staining. The migration positions of 5 S rRNA and tRNA are indicated.

The comparable affinities of the cysteine synthase and EcCysK:CdiA-CT complexes suggest that the toxin competes with EcCysE for access to EcCysK. We first used an indirect approach to test whether CdiA-CT interferes with the assembly of cysteine synthase complexes. Because EcCysE activity is stimulated when bound to EcCysK^55^, we titrated the CS complex with CdiA-CT and measured serine acetyltransferase activity. The maximal rate of serine acetylation was obtained with 28 nM EcCysE and 19 nM EcCysK, conditions in which the two proteins are at stoichiometric amounts based on the 3:2 CysE:CysK stoichiometry of the cysteine synthase complex. Pre-incubation of EcCysK with increasing concentrations of CdiA-CT reduced the stimulatory effect, decreasing acetyltransferase activity down to a plateau equivalent to that of free EcCysE (Fig. 3A).

We next examined the influence of EcCysE on the EcCysK:CdiA-CT complex, first testing whether excess EcCysE blocks toxin activation during CDI. We incubated target bacteria with inhibitor cells that deploy CdiA-CT, then isolated RNA from the mixed culture to detect toxic tRNase activity by Northern blot hybridization^60, 61^. To facilitate this analysis, we over-expressed tRNA_CCU_^Arg^ in the target-cell population. Because this substrate is present at very low levels in wild-type *E. coli* ^62^, essentially all of the tRNA_CCU_^Arg^ detected by Northern blot is derived from target bacteria. Most of the tRNA_CCU_^Arg^ substrate was cleaved within 1 h of co-culture (Fig. 3B, compare lanes 1 & 5); and this nuclease activity was dependent on EcCysK, because substrate was not degraded when ∆*cysK* mutants were used as target bacteria (Fig. 3B, lane 8). We then over-produced EcCysE in target cells and examined the effect on toxin activity. Notably, EcCysE was readily detected by SDS-PAGE analysis of crude lysates prepared from the co-culture (Fig. 3B, bottom panel), indicating that target cells likely contained enough EcCysE to saturate endogenous EcCysK. As predicted, over-produced EcCysE suppressed toxin activity, but substantial tRNA degradation was still detected in target cells (Fig. 3B, lane 6). By contrast, an EcCysE variant lacking 11 residues from the C-terminus was less effective in blocking toxin activity (Fig. 3B, lane 7), consistent with the importance of these residues in CS complex stability. We obtained similar results with *in vitro* tRNase assays. As reported previously^16^, CdiA-CT has no appreciable nuclease activity *in vitro*, but efficiently cleaves tRNA when reactions are supplemented with EcCysK (Fig. 3C, compare lanes 2 & 3). To examine the effect of EcCysE on nuclease activity, we pre-incubated EcCysK with EcCysE for 20 min to assemble cysteine synthase complexes. CdiA-CT was then added, and the protein mixture incubated for an additional 30 min prior to the addition of tRNA substrate. Even when used in eight-fold excess over EcCysK (with respect to cysteine synthase stoichiometry), EcCysE did not block tRNase activity to the same extent as CdiI immunity protein (Fig. 3C, compare lanes 6 & 7). Together, these results demonstrate that CdiA-CT toxin is activated efficiently even in the presence of EcCysE.

**Figure 4:**
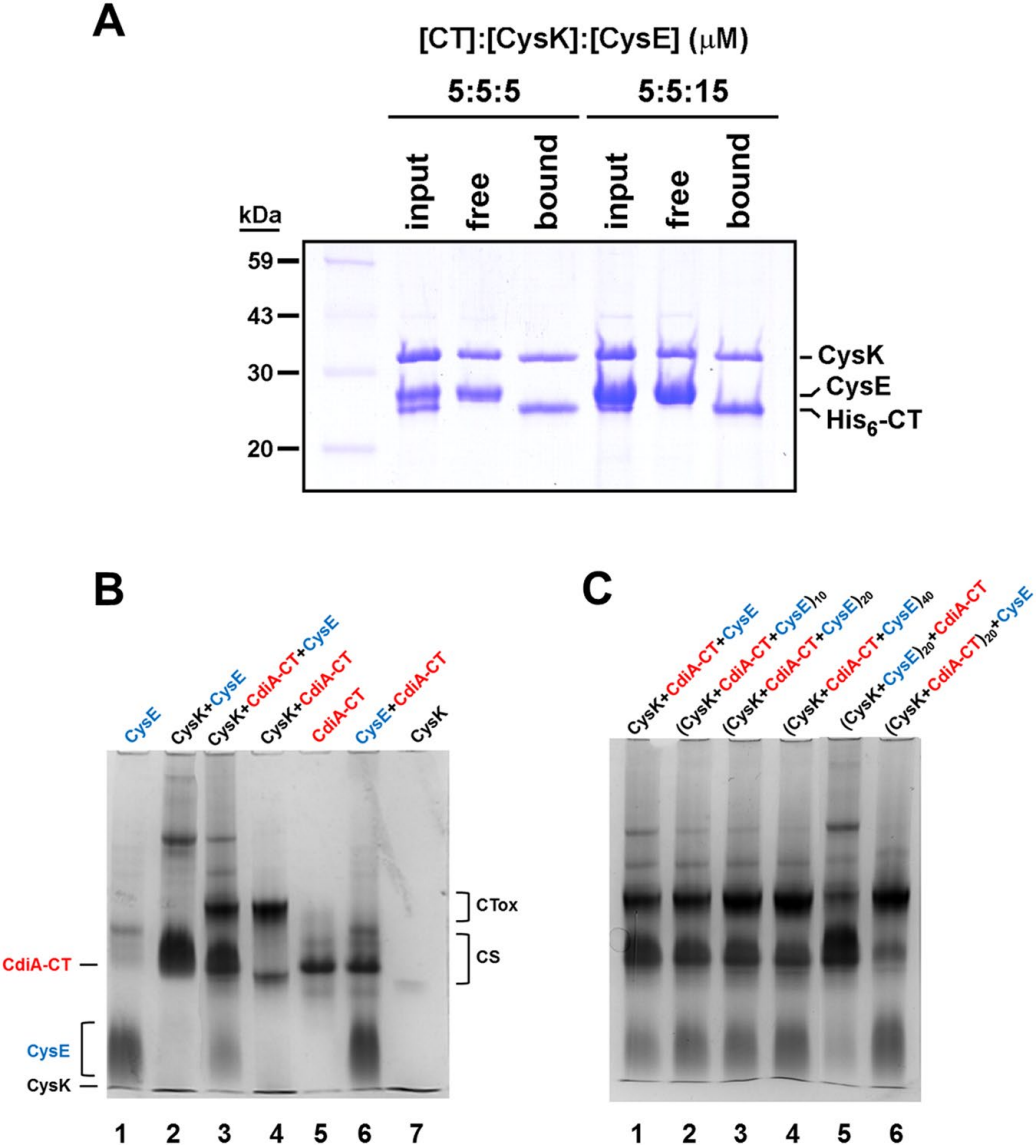
EcCysE and CdiA-CT compete for binding to CysK. (**A**) Affinity purification of EcCysK:CdiA-CT complexes. EcCysK, EcCysE and His_6_-tagged CdiA-CT were mixed at the indicated concentrations and subjected to Ni^2+^-affinity chromatography. The initial protein mixture (input), the column flow-through fraction (free) and the elution fraction (bound) were analyzed by SDS-PAGE. (**B**,**C**) Native PAGE analysis of EcCysK:EcCysE and EcCysK:CdiA-CT complexes. Proteins were mixed and run on non-denaturing polyacrylamide gels as described in the Methods. Proteins within parentheses were pre-incubated for 10, 20 or 40 min (as indicated by subscript) prior to native PAGE analysis. The migration positions of EcCysE, EcCysK and CdiA-CT are indicated on the left of panel B, and the migration positions of the cysteine synthase (CS) and EcCysK:CdiA-CT (CTox) complexes are indicated on the right.

The homodimeric structure of EcCysK provides a possible explanation for robust CdiA-CT activation in the presence of competing EcCysE. We reasoned that if only one EcCysK active site per dimer is occupied by EcCysE in the cysteine synthase complex, then the other active site should be available to bind toxin. This model predicts that EcCysK can bind EcCysE and CdiA-CT simultaneously. To explore this hypothesis, we sought to isolate EcCysE:EcCysK:CdiA-CT ternary complexes. We equilibrated His_6_-tagged CdiA-CT with untagged EcCysK and EcCysE for 1 h, then subjected the mixture to Ni^2+^-affinity chromatography to purify the toxin and associated proteins. EcCysK clearly interacted with His_6_-CdiA-CT under these conditions, but none of the EcCysE co-purified with the His_6_-CdiA-CT:EcCysK complex, even at concentrations up to 15 µM (Fig. 4A). Instead, there appeared to be competition for EcCysK occupancy, with much of the EcCysK remaining in the “free” fraction due to its association with EcCysE (Fig. 4A). These results show that high-affinity ternary complexes of EcCysE:EcCysK:CdiA-CT do not form, indicating that the binding of toxin and EcCysE to EcCysK is mutually exclusive.

We then developed a native PAGE approach to monitor the relative proportions of EcCysK:EcCysE and EcCysK:CdiA-CT in complex mixtures. Electrophoresis conditions were optimized to allow unambiguous identification of each complex based on its gel mobility (Fig. 4B, compare lanes 2 & 4). Simultaneous mixing of EcCysE, EcCysK and CdiA-CT resulted in the formation of both complexes, with lower levels (39%) of EcCysK:CdiA-CT with respect to CS (61%) (Fig. 4B, lane 3, Fig. 4C, lane 1). A similar result was obtained when EcCysE was pre-incubated with EcCysK (Fig. 4C, lane 5). By contrast, the proportion of CdiA-CT:EcCysK complex increased significantly (75%) when toxin and EcCysK were pre-incubated before the EcCysE addition (Fig. 4C, lane 6). This latter observation suggests that the CdiA-CT:EcCysK complex reaches equilibrium more slowly than CS. The finding is further supported by a time-driven experiment where EcCysE, EcCysK and CdiA-CT are mixed simultaneously and monitored over time. The proportion of EcCysK:CdiA-CT complex increased from about 40% to 60% after 40 min of incubation (Fig. 4C, lanes 1–4 and Supplemental Fig. S2). This result confirms that equilibrium conditions are reached slowly when EcCysE and CdiA-CT compete for EcCysK occupancy, and further suggests that CdiA-CT might displace EcCysE from the CS complex.

### CdiA-CT toxin is activated by CysK from diverse bacterial species

**Figure 5:**
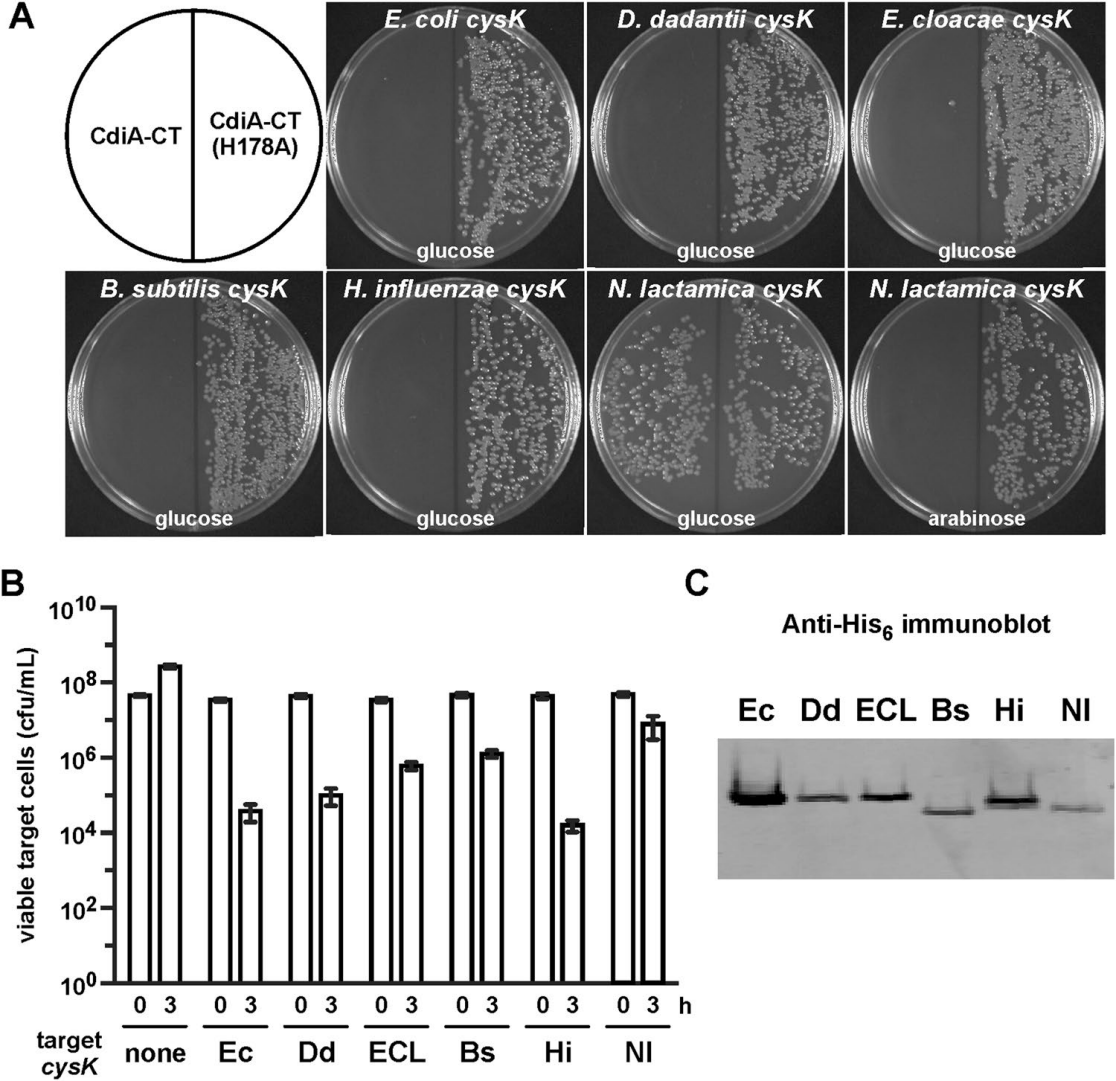
Heterologous CysK promotes CdiA-CT toxicity. (**A**) Activation of internally expressed CdiA-CT toxin. Plasmids encoding CdiA-CT and CysK proteins were introduced into *E. coli ∆cysK* cells, and transformants isolated on selective media supplemented with D-glucose or L-arabinose as indicated. To control for transformation efficiency, a plasmid encoding catalytically inactive CdiA-CT(H178A) was also tested. (**B**) CDI competition co-cultures. Inhibitor cells that deploy CdiA-CT were incubated with *E. coli ∆cysK* target bacteria that express CysK-His_6_ from the indicated bacterial species. Viable target bacteria were quantified as colony forming units per mL upon mixing and after 3 h of co-culture. Presented data are averages ± standard errors for four independent experiments. (**C**) Immunoblot analysis of heterologous CysK-His_6_. Total protein was isolated from the target-cell strains in panel B and analyzed by immunoblotting using antibodies to the His_6_ epitope. 10 μg of total protein was loaded in each lane.

CdiA-CT is a member of the Ntox28 RNase family and closely related toxin domains are found in CdiA proteins from *Enterobacter cloacae*, *Yersinia enterocolitica* and *Pseudomonas syringae* ^14, 15, 42, 43^. These observations suggest that CdiA-CT interacts with other CysK enzymes to ensure activation in different bacterial species. We tested this prediction using a previously described plasmid-transformation assay^16, 60^. In this assay, separate plasmids that express CdiA-CT or CysK are simultaneously introduced into *E. coli ∆cysK* cells, and transformants are selected on antibiotic-supplemented media. Because CdiA-CT is toxic when bound to EcCysK, cells that take up both plasmids are unable to grow, and therefore stable transformants are not obtained even when toxin expression is repressed with D-glucose in the media (Fig. 5A). To control for transformation efficiency, we introduced a catalytically inactive CdiA-CT construct carrying the His178Ala mutation and obtained several transformants (Fig. 5A)^16^. We then tested plasmids encoding heterologous enzymes that share between 50% and 96% sequence identity with EcCysK. These CysK homologs share virtually identical active sites, and 8 of the 13 residues that make direct contact with the toxin domain are conserved (Fig. S3). As expected, closely related enzymes from *Enterobacter cloacae* (ECLCysK, 96% identity) and *Dickeya dadantii* (DdCysK, 91% identity) promoted CdiA-CT toxicity in the transformation assay (Fig. 5A). More distantly related CysK proteins from *Haemophilus influenzae* (HiCysK, 68% overall identity, 92% identity in toxin-binding residues) and *Bacillus subtilis* (BsCysK, 50% overall identity, 92% identity in toxin-binding residues) also activated the toxin *in vivo* (Fig. 5A). However, NlCysK from *Neisseria lactamica* (53% identity, 69% identity in toxin-binding residues) only supported toxicity when its expression was fully induced with L-arabinose (Fig. 5A, compare glucose and arabinose plates). This latter result suggests that CdiA-CT has significantly lower affinity for NlCysK. Similar results were obtained when we tested the function of heterologous CysK in CDI competition co-cultures. We provided *E. coli ∆cysK* target cells with *cysK-his*_6_ expression plasmids and incubated the resulting strains with inhibitor bacteria that deploy CdiA-CT. Growth inhibition was assessed by enumerating viable target bacteria after three hours of co-culture. Target cells lacking CysK were resistant to growth inhibition, and viable cell counts increased during the incubation (Fig. 5B). By contrast, target cells expressing EcCysK, DdCysK and HiCysK were inhibited, with each population showing ~100-fold losses in viability (Fig. 5B). The other CysK enzymes were less effective at promoting toxicity, particularly NlCysK, which showed less than a ten-fold decrease in viable cell counts (Fig. 5B). To ascertain the levels of heterologous CysK in target cells, we performed immunoblot analysis using antibodies to the His_6_ epitope appended to the C-terminus of each enzyme. This analysis revealed lower levels of BsCysK and NlCysK (Fig. 5C), perhaps accounting for the resistance of NlCysK expressing cells to growth inhibition.

**Figure 6:**
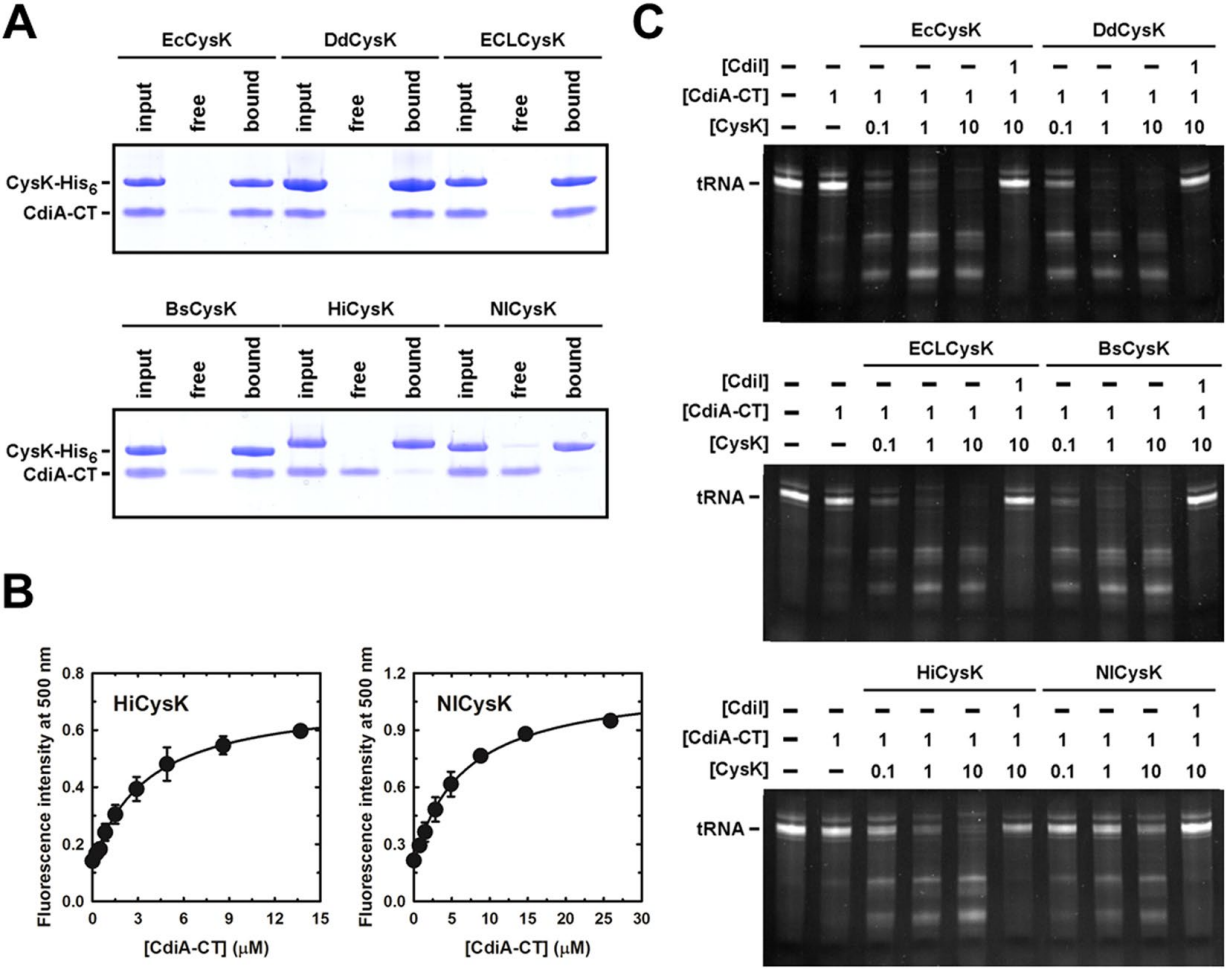
*In vitro* activation of CdiA-CT by heterologous CysK. (**A**) Affinity purification of CysK:CdiA-CT complexes. His_6_-tagged CysK proteins from the indicated species were incubated with untagged CdiA-CT and the mixture subjected to Ni^2+^-affinity chromatography. The initial protein mixture (input), the column flow-through fraction (free) and the elution fraction (bound) were analyzed by SDS-PAGE. (**B**) Determination of the CysK:CdiA-CT dissociation constants. HiCysK (300 nM) and NlCysK (860 nM) were titrated with increasing concentrations of CdiA-CT, and fluorescence emission at 500 nm monitored after 20 min. Eq. 1 was fitted to the binding data to obtain *K*_d_ = 3.3 ± 0.3 µM for HiCysK and *K*_d_ = 6.4 ± 0.6 µM for NlCysK. (**C**) Heterologous CysK promotes CdiA-CT nuclease activity. Proteins were incubated at the indicated concentrations (µM) for 20 min prior to the addition of RNA substrate. Reactions were quenched after 1 h at 37 °C, then run on 8 M urea-polyacrylamide gels and visualized by ethidium bromide staining. The migration position of full-length tRNA is indicated.

Finally, we examined toxin binding and activation by heterologous CysK *in vitro*. We first used affinity co-purification to screen interactions between CdiA-CT and His_6_-tagged CysK proteins. This approach showed that DdCysK, ECLCysK and BsCysK all form high-affinity complexes with CdiA-CT (Fig. 6A). Because the toxin failed to co-purify with HiCysK and NlCysK (Fig. 6A), we quantified the binding interactions using fluorimetric titrations and determined dissociation constants of 3.3 ± 0.3 µM for the HiCysK:CdiA-CT complex and 6.4 ± 0.6 µM for NlCysK:CdiA-CT (Fig. 6B). Thus, CdiA-CT binds these latter enzymes with ~1,000-fold lower affinity than EcCysK. Consistent with this low affinity, high concentrations of HiCysK and NlCysK were required to activate the CdiA-CT nuclease *in vitro* (Fig. 6C). As we found in the *in vivo* analyses, NlCysK was the least effective at promoting toxin activity. In fact, tRNase reactions supplemented with NlCysK up to 10 µM did not go to completion after 1 h incubation. Together, these results show that CdiA-CT toxin can be activated by a variety of CysK enzymes, but the binding constants span several orders of magnitude.

## Discussion

Here, we show that EcCysK and CdiA-CT form a high-affinity complex with two toxin domains bound per EcCysK homodimer. These results are broadly consistent with a prior thermodynamic study by Kaundal *et al*.^57^, though our data indicate that CdiA-CT could displace EcCysK from pre-formed CS complexes and suggest that CdiI has less of an effect on binding affinity. However, both studies show that complex formation is remarkably slow. Kaundal *et al*. used surface plasmon resonance to measure a k_on_ of 6.2·10^3^ M^−1^·s^−1^ ^57^, and here we calculate a second-order rate constant of 2·10^4^ M^−1^·s^−1^ for EcCysK:CdiA-CT binding, consistent with slow conformational rearrangements following the formation of an encounter complex. The cysteine synthase complex also exhibits a two-step binding mechanism^56^, which is perhaps not surprising given that the CdiA-CT toxin mimics CysE by inserting its C-terminus into the CysK active site, anchoring the interaction to allow further conformational changes. For the CS complex, the slow conformational changes entail closure of the CysK active site^38, 56^; but allosteric changes in CysE are also likely because its *O*-acetyltransferase activity is stimulated in the *E. coli* complex (see Fig. 3A). We note that EcCysK adopts an open active-site conformation in the EcCysK:CdiA-CT crystal structure^42^, indicating that the toxin does not induce significant structural changes in EcCysK. Together, these observations suggest that conformational changes in CdiA-CT are responsible for the slow phase of complex formation. This model also accounts for toxin activation, whereby EcCysK-induced structural changes organize the nuclease active site for catalysis. CdiA-CT is probably delivered in a partially unfolded state (vide infra) and folding to the final active conformation likely represents the slow, rate limiting step in complex formation. Reversal of this isomerization, which is described by the k_4_ rate constant is even slower (see Fig. 2E). Direct determination of k_4_ is hindered by the intrinsically high error in the calculation of the y-axis intercept. However, Eq. 8 can be used to estimate the rate constant for the reversal of isomerization at 2.2·10^−4^ s^−1^, which is two orders of magnitude slower than the corresponding value of 0.024 s^−1^ for the CS complex^56^ and in good agreement with the overall k_off_ calculated by SPR^57^. Thus, the activated toxin complex dissociates exceptionally slowly, prolonging nuclease activity in target cells. Such long residence times have been observed for antigen-antibody and protease-inhibitor interactions and are important in open biological systems, where ligand concentrations vary over time^63^. For many protein-ligand interactions, binding efficacy can be explained entirely by the k_off_ value rather than dissociation constant alone^64^.

**Figure 7:**
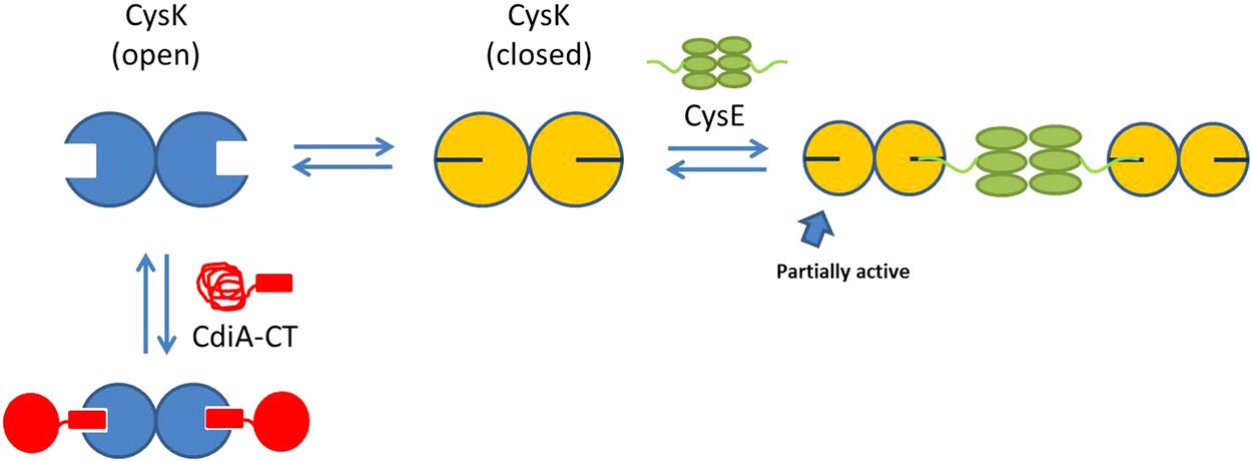
Formation of CS and CysK:CdiA-CT complexes. CysK exists in two conformations: an open, inactive conformation and a closed one that was isolated as a complex with substrate analog^31^. Structural data^42^ demonstrate that CdiA-CT binds to the open conformation of CysK. Binding is slow and is limited by a large conformational change, which likely corresponds to CdiA-CT folding to its active conformation. Here, we propose that CysE preferentially binds to CysK in the closed active-site conformation.

EcCysE and CdiA-CT bind with comparable affinities to the same site on EcCysK, indicating that the toxin must compete with EcCysE in order to be activated. Further, the toxin is presumably at a disadvantage with respect to endogenous EcCysE, because only a few CdiA-CT domains are delivered into target cells during CDI^65^. This is compounded by the fact that the second-order rate constant for CS complex formation is ~200-fold greater than that of the EcCysK:CdiA-CT complex. However, early studies in *Salmonella* Typhimurium suggested that StCysK levels exceed those of StCysE, with only 5–25% of StCysK found in the CS complex^66, 67^. On the other hand, *cysE* and *cysK* are regulated by different transcription factors, raising the possibility that their relative proportions are modulated in response to changing growth conditions. For example, high cysteine levels inhibit CysE activity^68, 69^, reducing the production of *O-*acetyl-L-Ser and *N-*acetyl-L-Ser, which are required as co-activators to induce CysB-dependent transcription of the *cys* regulon^17^. Because *cysE* transcription is not regulated by CysB, it is possible that CysE becomes more abundant than CysK when the cell is replete with cysteine. Moreover, recent transcriptomic data show that *cysK* and *cysE* transcript levels are comparable in *S*. Typhimurium cells grown in rich media and other conditions^70^. Thus, CdiA-CT activity and CDI could be modulated by environmental conditions, though we have found that target bacteria are still inhibited in cysteine supplemented media (C.M.B. & C.S.H., unpublished data). These observations indicate that EcCysE levels are no impediment to toxin activation. Moreover, the data presented here show that even supra-physiological EcCysE concentrations are insufficient to block toxin activation. Thus, CdiA-CT competes effectively with EcCysE, and may even displace EcCysK from pre-formed CS complexes. There are no structures available for the CS complex, but biochemical studies indicate that each CysE hexamer engages two CysK dimers (Fig. 7). Further, molecular modeling shows that distance and geometrical constraints prevent CysE from engaging both CysK active sites simultaneously^71^. Therefore, only one active site per CysK dimer is engaged with CysE in the CS complex (Fig. 7). This architecture provides opportunities for CdiA-CT to bind the unoccupied CysK active site. However, stable EcCysE:EcCysK:CdiA-CT ternary complexes cannot be isolated, suggesting that the binding of CdiA-CT and EcCysE to EcCysK is mutually exclusive. Several studies indicate that the CysK active site undergoes allosteric closure in the CS complex^38, 56^, whereas CdiA-CT binds to EcCysK with an open active-site conformation (Fig. 7)^42^. Thus, differential affinities for the open and closed states could account for the observed binding behavior. This model may also explain how CdiA-CT disrupts the CS complex, though we note that because the two complexes have similar affinities, their proportions at equilibrium should reflect the relative concentrations of toxin and EcCysE.

The CdiA-CT toxin from *E. coli* 536 has evolved a dependence on CysK, but most other CDI toxins do not require additional factors to promote toxicity^12, 13, 72–74^. Moreover, the benefit of extrinsic activation is not clear in the context of inter-bacterial conflict, because target bacteria can readily acquire resistance through *cysK* mutations^16, 60^. One explanation invokes possible physical constraints on CDI toxin delivery, which entails CdiA-CT translocation across the outer and inner membranes of target bacteria. Though the mechanistic basis of CDI toxin transfer is not completely understood, the analogous import of colicins into *E. coli* requires the unfolding of toxin domains^75, 76^. If CDI toxins must also unfold during delivery, then there should be a selective pressure for domains with low global stability. This in turn could provide the impetus to evolve binding interactions that compensate for intrinsic instability. Consistent with this hypothesis, CdiA-CT has relatively low thermostability and is significantly stabilized when bound to EcCysK^42, 57^. Thus, EcCysK-binding could ensure that the toxin regains its native fold after delivery into the target-cell cytoplasm. In principle, the CdiA-CT toxin could have evolved binding interactions with any number of cytosolic proteins, but it appears that CysK was selected due to its conservation throughout bacteria. Although uropathogenic *E. coli* are unable to deliver the CdiA-CT toxin into other bacterial species^45, 46^, there is still a selective pressure for activation in diverse bacteria because CDI systems are encoded on mobile genetic elements and are spread by horizontal gene transfer^47, 48^. Database searches reveal closely related toxin domains (>60% sequence identity) in CdiA proteins from various Enterobacteriaceae and Pseudomonads. By targeting the conserved active-site cleft of CysK, the toxin is likely to be activated in the cytosol of any given species. Finally, we note that this phenomenon appears to be widespread, because unrelated CDI toxins from *E. coli* isolates EC869, NC101 and 96.154 have recently been shown to interact functionally with the highly conserved translation factors EF-Tu and EF-Ts^77^.

## Methods

### Bacterial strains and plasmid constructions

Bacterial strains and plasmids are listed in Table 1. Bacteria were grown in lysogeny broth (LB) or on LB agar unless otherwise noted. Where indicated, media were supplemented with antibiotics at the following concentrations: ampicillin, 150 µg mL^−1^; kanamycin, 50 µg mL^−1^; rifampicin, 200 µg mL^−1^; and tetracycline, 12.5 µg mL^−1^. The *∆cysK::kan* disruption was obtained from the Keio collection^78^ and transduced into *E. coli* strains MG1655 (DE3) and CH10013. Kanamycin-resistance cassettes were subsequently removed with FLP recombinase expressed from plasmid pCP20^79^. Bacterial *cysK* open-reading frames were amplified by PCR using the following primer pairs: CH2095/CH2102 for *D. dadantii* 3937, CH2101/CH2099 for *E*. *cloacae* ATCC 13047, CH3466/CH3467 for *H. influenzae* Rd, CH3345/CH3346 for *N. lactamica* ATCC 23970, and CH2096/CH2094 for *B. subtilis* 168 (Table S1). The resulting products were digested with NcoI/SpeI, then ligated to plasmid pCH6505 to generate T7 over-expression constructs, and to plasmid pCH6478 for complementation of *E. coli ∆cysK* mutants. The *N. lactamica cysK* fragment was ligated using NcoI/XhoI restriction sites. *E. coli cysE* was amplified with primers CH3642/CDI235 and ligated to pET21P with KpnI/XhoI restiction sites to generate pCH12028 for the gratuitous over-production of native EcCysE in target cells. This fragment was also ligated to pCH10068^80^ to generate plasmid pCH9764 for the purification of untagged EcCysE. Primers CH3642/CH4125 were used to generate plasmid pCH13299, which over-produces EcCysE lacking 11 residues from the C-terminus. The *E. coli cysK* gene was amplified with primers CH3865/CH2797 and ligated to pCH10068 for the purification of untagged EcCysK.

**Table 1:** Bacterial strains and plasmids.

**Strain or plasmid**	**Description** ^**a**^	**Reference**
***Bacterial strains***		
BL21 Tuner^TM^ (DE3)	*E. coli* B, F^−^ *ompT hsdS*(r_B_^−^ m_B_^−^) *dcm* ^+^ *gal* λ(DE3) *endA ∆lacZY* Hte, Tet^R^	Novagen
EPI100	F^−^ *mcrA ∆(mrr-hsdRMS-mcrBC) φ80dlacZ∆M15 ∆lacXcZ∆M15 ∆lacX recA1 endA1 araD139 ∆(ara, leu)7*6*97 galU galK* λ^*–*^ *rpsL nupG*, Str^R^	Epicentre
CH2016	X90 (DE3) ∆*rna ∆slyD::kan*, Rif^R^ Kan^R^	81
CH7076	MG1655 (DE3)	This study
CH7718	X90 (DE3) *∆rna ∆cysE::kan*, Rif^R^ Kan^R^	This study
CH8804	X90 (DE3) *∆rna ∆slyD* ∆*cysK::kan*, Rif^R^ Kan^R^	42
CH10028	JCM158 ∆*cysK::kan*, Rif^R^ Kan^R^	This study
CH10801	JCM158 ∆*cysK*, Rif^R^	This study
CH13316	MG1655 (DE3) ∆*cysK::kan*, Kan^R^	This study
***Plasmids***		
pTrc99a	IPTG-inducible expression plasmid, Amp^R^	GE Healthcare
pET21P	T7 RNA polymerase expression plasmid, Amp^R^	11
pCP20	Heat-inducible expression of FLP recombinase, Cm^R^ Amp^R^	79
pDAL866	Arabinose-inducible expression of the *E. coli cdiBAI* gene cluster, Cm^R^ Amp^R^	65
pCH450	pACYC184 derivative with *E. coli araBAD* promoter for arabinose-inducible expression, Tet^R^	82
pCH1043	pCH405∆::*argW*, over-expresses tRNA_CCU_^Arg^, Tet^R^	83
pCH6190	pET21P::*cdiA-CT/cdiI*, over-produces CdiA-CT and CdiI-His_6_, Amp^R^	11
pCH6478	pTrc99A::*cdiA-CT3/cdiI3* ^Dd3937^-*his*6, Amp^R^	47
pCH6505	pET21S::*cdiA-CT/cdiI* ^Dd3937^, Amp^R^	11
pCH7086	pCH450::*cdiA-CT(H178A)*, Tet^R^	16
pCH8215	pET21S::*Ec-cysK*, Amp^R^	16
pCH8936	pET21S::*Dd-cysK*, Amp^R^	This study
pCH8937	pET21S::*ECL-cysK*, Amp^R^	This study
pCH8639	pET21S::*Bs-cysK*, Amp^R^	This study
pCH9280	pTrc99A::*Ec-cysK-his*_6_, Amp^R^	16
pCH9320	pCH450::*cdiA-CT*, Tet^R^	16
pCH9764	pSH21::*trxA-TEV-cysE*, Amp^R^	This study
pCH10068	pSH21::*trxA-TEV-rhs-CT*(H208A)*-rhsI*, Amp^R^	80
pCH11846	pET28a(+)::*Hi-cysK*, over-produces CysK-His_6_ from *Haemophilus influenzae*, Kan^R^	56
pCH11860	pTrc99A::*Nl-cysK-his*_6_, Amp^R^	This study
pCH12028	pET21P::*Ec-cysE*, Amp^R^	This study
pCH12113	pTrc99A::*Hi-cysK-his*_6_, Amp^R^	This study
pCH12114	pET21S::*Nl-cysK*, Amp^R^	This study
pCH12146	pET21S::*Hi-cysK*, Amp^R^	This study
pCH12286	pTrc99A::*Dd-cysK-his*_6_, Amp^R^	This study
pCH12287	pTrc99A::*ECL-cysK-his*_6_, Amp^R^	This study
pCH12288	pTrc99A::*Bs-cysK-his*_6_, Amp^R^	This study
pCH10673	Expresses chimeric CdiA^EC93^-CT^EC536^, Cm^R^	84
pCH12618	pSH21::*trxA-TEV-cysK*, Amp^R^	This study
pCH13129	pET21::*his*_6_ *-cdiA-CT(H178A)*, Amp^R^	42
pCH13299	pET21P::*Ec-cysE(∆I263-I273)*, Amp^R^	This study

^a^Abbreviations: Amp^R^, ampicillin-resistant; Cm^R^, chloramphenicol-resistant; Kan^R^, kanamycin-resistant; Rif^R^, rifampicin-resistant; Str^R^, streptomycin-resistant; Tet^R^, tetracycline-resistant.

### Protein expression and purification

Proteins were over-produced in *E. coli* BL21(DE3) Tuner^TM^ or CH2016 cells grown in LB media supplemented with 1 mM isopropyl β-D-1-thiogalactopyranoside (IPTG). Cells were resuspended in buffer A [20 mM sodium phosphate (pH 7.0), 85 mM sodium chloride, 10 mM 2-mercaptoethanol, 2 mM EDTA] and broken by sonication or French pressure cell. His_6_-tagged proteins were purified by Ni^2+^- or Co^2+^-affinity chromatography according to^85^ with minor modifications. The His_6_ epitope tag was removed from HiCysK using thrombin^36^. CysK concentrations were determined by PLP absorbance, calculated by the alkali denaturation method^86^. Extinction coefficients at 412 nm are 7,600 M^−1^·cm^−1^ for HiCysK, 9,370 M^−1^·cm^−1^ for EcCysK, and 8,280 M^−1^·cm^−1^ for NlCysK. Purity was assessed by SDS-PAGE and demonstrated to be greater than 97% (Fig. S4A). All enzymes showed the typical absorption spectrum of fold-type II PLP-dependent enzymes with peaks at 278 and 412 nm and a specific activity of 0.025 U/mg, 0.013 U/mg and 0.016 U/mg for EcCysK, HiCysK and NlCysK (respectively) in agreement with previously reported kinetic data^23^.

EcCysE was over-produced as a fusion with His_6_-thioredoxin (His_6_-TrxA) linked by a TEV protease recognition sequence. Affinity resins were washed with buffer containing 10 mM *O*-acetyl-L-Ser to dissociate contaminating EcCysK. His_6_-TrxA-EcCysE was eluted with 1 M imidazole and dialyzed against 20 mM Tris-HCl (pH 7.5), 50 mM NaCl, 1% glycerol, 1 mM dithiothreitol, 1 mM EDTA. The fusion was digested with His_6_-tagged TEV protease for 4 h at 25 °C, and the His_6_-TrxA and His_6_-TEV proteins were removed by metal-affinity chromatography. EcCysE was loaded on a FPLC column packed with Ultrogel AcA44 resin (exclusion limit 200 kDa, operating range 17–175 kDa, column volume 63 mL and void volume 20.4 mL) and run at 0.2 mL/min in buffer A. EcCysE eluted at 28 mL with an apparent molecular mass of 167,200 Da, consistent with the expected hexameric quaternary structure. Protein concentration was calculated using an extinction coefficient at 278 nm of 26,900 M^−1^ cm^−1^. Purified EcCysE was ~96% pure (Fig. S4A), with a specific activity of 290 U/mg in agreement with previously published reports^87, 88^.

The CdiA-CT:CdiI-His_6_ complex was expressed in either *E. coli* BL21(DE3) Tuner^TM^ or CH2016 as described^11^. CdiA-CT and CdiI-His_6_ were separated by metal-affinity chromatography in 8 M urea and the proteins refolded by dialysis into buffer A. The isolated proteins were greater than 95% pure (Fig. S4A), and circular dichroism spectroscopy showed that each protein regained native structure under these conditions (Fig. S4B). CdiA-CT was further purified using size-exclusion chromatography as described for EcCysE above. Protein concentration was estimated using an extinction coefficient at 278 nm of 13,300 M^−1^·cm^−1^ and 8,480 M^−1^·cm^−1^ for CdiA-CT and CdiI, respectively.

### Spectroscopy

Absorption spectra were collected at 20.0 ± 0.5 °C using a Varian CARY400 spectrophotometer. All spectra were corrected for buffer contributions. Circular dichroism measurements were carried out using a JASCO J-715 spectropolarimeter. Each spectrum is the average of three measurements and is subtracted of the buffer contribution. EcCysE/CdiA-CT binding to CysK was monitored by measuring PLP fluorescence emission at 500 nm following excitation at 412 nm^38, 56^. CysK emission spectra were collected using a FluoroMax-3 fluorometer (HORIBA) at 20 ± 0.5 °C. Unless otherwise specified, titration samples were equilibrated for 5 min prior to spectra acquisition. All spectra were corrected for buffer contribution, and the slit width set to optimize the signal to noise ratio. For equilibrium binding experiments, the dependence of emission intensity on ligand concentration was determined using the binding isotherm:

1 $$I={I}_{0}\cdot \frac{{I}_{{\rm{m}}{\rm{a}}{\rm{x}}}\cdot [L]}{{K}_{d}+[L]}$$

or a quadratic equation that describes tight binding:

2 $$I={I}_{0}+{I}_{{\rm{m}}{\rm{a}}{\rm{x}}}\cdot \frac{[CysK]+[CdiA-CT]+{K}_{d}-\sqrt{{([CysK]+[CdiA-CT]+{K}_{d})}^{2}-4\cdot [CysK]\cdot [CdiA-CT]}}{2}$$

where *I* is the fluorescence intensity at 500 nm, *I*_0_ is a horizontal offset, *I*_max_ is the maximum change in fluorescence at saturating [CdiA-CT] and *K*_d_ is the dissociation constant for the CysK:CdiA-CT complex.

### Enzyme activity assays

CysK specific activities were quantified by a continuous spectrophotometric assay using 2-thio-5-nitrobenzoate (TNB) as a nucleophilic substrate^23^. EcCysE specific activity was determined indirectly with 5,5′-dithio-bis(2-nitrobenzoic acid) (DTNB) as described^89^. An extinction coefficient of 14,150 M^−1^·cm^−1^ at 412 nm was used to quantify TNB in both enzyme assays^90^. EcCysE steady-state kinetics were measured by an adaptation of a published method^91^ in buffer A without 2-mercaptoethanol at 20 °C. Briefly, *O*-acetylation of 20 mM L-Ser was monitored by measuring the absorption at 232 nm of the thioester bond (ε_232_ = 4,440 M^−1^ cm^−1^), while varying acetyl-CoA concentrations. At a fixed 0.25 mM acetyl-CoA concentration, EcCysE activity increases as a function of EcCysK concentration^55^. Displacement of EcCysE from the cysteine synthase complex by CdiA-CT was monitored using 28 nM CysE (4.7 nM hexamer) in the presence of 19 nM CysK (9.5 nM dimer). EcCysK and CdiA-CT were incubated for 20 min at 20 °C, then EcCysE and L-Ser were added, and the reaction was initiated with the addition of acetyl-CoA.

CysK steady-state kinetics were measured by quantifying L-Cys using the discontinuous method of Gaitonde in a 96-well plate format^92^. The sulfhydrylase reaction was initiated by addition of 0.6 mM Na_2_S to a solution containing 6 nM EcCysK, 60 nM bovine serum albumin and variable concentrations of *O*-acetyl-L-Ser in buffer A. Aliquots (60 µL) were removed at intervals and quenched with 60 µL of acetic acid in a PCR tube strip. Ninhydrin (60 µL) was added with a multichannel pipette and the mixture heated at 100 °C for 10 min in a thermal cycler. The solution was cooled down and 46 µL were added to the wells of a 96-well plate containing 154 µL of cold ethanol. The absorbance at 550 nm was measured using a plate reader and blanks subtracted. The amount of L-Cys produced at each time point was calculated from a calibration curve and a linear equation was fitted to the data to determine initial rate (*v*_*i*_) of production. All kinetic data sets were collected from at least two independent experiments. The kinetic parameters were calculated as follows: k_cat_ = 241 ± 5 s^−1^, K_M,OAS_ = 5.1 ± 0.3 mM and K_M,HS_^−^ = 0.006 ± 0.003 mM. The dependence of the initial velocity on either EcCysE or CdiA-CT concentration was measured in buffer A containing 2 mM *O*-acetyl-L-Ser. Morrison’s equation (3) was used to calculate *IC*_50_ and hence the *K*_i_ for tight-binding inhibitors^63^:

3 $$\frac{{v}_{i}}{{v}_{0}}=1-\frac{({[E]}_{T}+{[I]}_{T}+I{C}_{50})-\sqrt{{({[E]}_{T}+{[I]}_{T}+I{C}_{50})}^{2}-4\cdot {[E]}_{T}\cdot {[I]}_{T}}}{2\cdot {[E]}_{T}}$$

where [E]_T_ is the total enzyme concentration, and [I]_T_ is the total inhibitor concentration (EcCysE or CdiA-CT). For competitive inhibitors of a ping-pong reaction^63, 93, 94^:

4 $$I{C}_{50}={K}_{i}\cdot [1+\frac{[OAS]}{{K}_{M,OAS}}\cdot (1+\frac{{K}_{M,H{S}^{-}}}{[H{S}^{-}]})]$$

### Pre-steady state binding kinetics

Pre-steady state kinetic traces were collected under similar conditions to those reported in ref. 56. Experiments were carried out in buffer A under pseudo-first order conditions with 200 nM EcCysK, 270 nM CdiA-CT or 400 nM EcCysE. The temperature of the loading syringes and the stopped-flow cell compartment was maintained constant with a circulating water bath. Kinetic traces were collected upon direct excitation of PLP at 412 nm using an SX-18MV apparatus (Applied Photophysics) equipped with a 75-watt xenon lamp as a light source and a photomultiplier as a detector. The emission signal was collected at 90° with respect to the excitation source and filtered below 440 nm by a cut-off filter. A single exponential equation

5 $${I}_{t}={I}_{0}+I\cdot {e}^{(\frac{t-{t}_{0}}{\tau })}$$

was fitted to data averaged from three to five kinetic traces. *I*_t_ and *I*_0_ are the emission values at a given time and at zero time, respectively; *I* is the total fluorescence change, and τ is the relaxation time, such that *k*_obs_ is 1/τ. The dependence of k_obs_ on protein concentrations was obtained from the linear equation:

6 $${k}_{obs}={k}_{4}+(\frac{{k}_{3}}{{K}_{d}})\cdot [P]$$

to account for a two-step mechanism with a slow conformational change where the plateau cannot be attained under the experimental conditions^56, 59^.The *K*_d_ in Eq. 6 accounts for the first step (i.e. encounter complex formation) of a two-step binding reaction. The dissociation constant as measured under equilibrium conditions accounts for the contributions of both binding and isomerization steps and is usually indicated as ${K}_{d}^{overall}$, indeed for a slow binding mechanism, where k_4_<<k_3_^56, 59^:

7 $${K}_{d}^{overall}={K}_{d}\cdot (\frac{{k}_{4}}{{k}_{3}})$$

If k_3_/*K*_d_ and ${K}_{d}^{{overall}}$ are known, then k_4_ can be calculated as follows:

8 $${k}_{4}={K}_{d}^{overall}\cdot (\frac{{k}_{3}}{{K}_{d}})$$

### Complex co-purification and native gel electrophoresis

Purified EcCysK (5 µM) and His_6_-tagged CdiA-CT (5 µM) were incubated with EcCysE (5 or 15 µM) in 20 mM sodium phosphate (pH 7.5), 140 mM NaCl for one h at room temperature. A sample of the mixture was removed (for subsequent SDS-PAGE analysis) and the remainder subjected to Ni^2+^-affinity chromatography as described^16^. Samples of the original mixture (input), the column void (free) and imidazole elution (bound) were analyzed by SDS-PAGE and proteins detected with Coomassie blue stain. The same procedure was used to screen for stable interactions between bacterial CysK-His_6_ proteins and untagged CdiA-CT. Native gel electrophoresis was used to detect cysteine synthase and activated toxin complexes in mixtures. Purified EcCysK (12 µM monomer), EcCysE (18 µM monomer) and CdiA-CT (12 µM) were mixed in various combinations and the resulting complexes resolved on 8% polyacrylamide gels run at 10 mA constant current and 4 °C. The gel running buffer was 5 mM sodium phosphate (pH 7.0) and proteins were detected with Coomassie blue stain. Native-PAGE gels were analyzed using Image Lab™ software (version 5.2.1, Bio-Rad). Software auto analysis procedure was applied to detect lanes and bands with manual adjustments. The exposure time was set to 0.074 s. The relative intensity of each band was calculated using the band % parameter, which calculates band volume as percentage of the total band volume for each sample lane.

### Competition co-cultures and *in vivo* toxin activity

*E. coli* EPI100 cells that deploy CdiA-CT from plasmid pCH10673 were used as inhibitors in experiments to determine the effect of EcCysE over-production on toxin activation in target bacteria. Inhibitors were mixed at a 1:1 ratio with *E. coli* CH7076 (*cysK* ^+^) or CH13316 (∆*cysK*) target cells that overexpress tRNA_CCU_^Arg^. Target cells also harbored plasmids pET21P, pCH12028 or pCH13299 (where indicated), and were induced with 1 mM IPTG to allow EcCysE accumulation for 30 min prior to mixing with inhibitor cells. Samples were harvested into an equal volume of ice-cold methanol upon initial cell mixing and after 1 h of co-culture. Cells were collected by centrifugation at 4 °C and frozen at −80 °C. RNA was extracted from frozen cell pellets with guanidinium isothiocyanate-phenol as described previously^81^. RNA was resolved on 50% urea – 10% polyacrylamide gels and electro-blotted to nylon membrane and hybridized to 5′-radiolabeled oligonucleotide (5′ – CCT GCA ATT AGC CCT TAG G)^83^. Protein was isolated from co-culture samples with two freeze-thaw cycles in urea lysis buffer [8 M urea, 50 mM Tris-HCl (pH 8.0), 150 mM NaCl]. Urea-soluble protein was quantified by Bradford assay and 10 µg resolved on SDS-polyacrylamide gels. Proteins were detected with Coomassie blue stain.

Plasmid co-transformation was used to assess CdiA-CT toxicity in combination with heterologous CysK enzymes. Arabinose-inducible CdiA-CT expression plasmids (100 ng) were introduced into *E. coli ∆cysK* cells together with plasmids pTrc99A (no CysK), pCH9280 (EcCysK), pCH11860 (NlCysK), pCH12113 (HiCysK), pCH12286 (DdCysK), pCH12287 (ECLCysK) or pCH12288 (BsCysK). After recovery for 1 h at 37 °C in LB media supplemented with 0.4% D-glucose, cells were plated onto LB-agar supplemented with Tet, Amp and 0.4% D-glucose or L-arabinose to select for transformants carrying both plasmids.

*E. coli* EPI100 inhibitors that express the *cdiBAI* ^EC536^ gene cluster from pDAL866 were used in competition co-cultures to test complementation with heterologous *cysK*. *E. coli* CH10801 (∆*cysK*) target cells harboring the various *cysK* expression plasmids were grown to mid-log phase in LB media supplemented with ampicillin, then mixed at a 1:10 ratio with inhibitor cells in LB medium supplemented with 0.2% L-arabinose and incubated for 3 h at 37 °C with vigorous shaking in baffled flasks. Viable target-cell counts were enumerated as colony forming units (cfu) mL^−1^ on LB-agar supplemented with rifampicin. Data are presented as averages ± standard errors for four independent experiments. Heterologous CysK levels were monitored by immunoblot analysis. Total protein was isolated from target-cell strains using urea lysis as described above. Proteins were resolved by SDS-PAGE, electro-blotted onto nitrocellulose, and CysK detected with polyclonal antibodies to the C-terminal His_6_ epitope. Immunoblots were visualized using IRDye^®^ 680 (LI-COR) labeled anti-rabbit secondary antibodies and an Odyssey^®^ infrared imager as described previously^95^.

### *In vitro* nuclease assays

CdiA-CT tRNase activity was assayed in 20 mM Tris-HCl (pH 7.5), 150 mM NaCl, 0.5 mM MgCl_2_ at 37 °C. To determine the influence of EcCysE on nuclease activity, EcCysK (0.5 µM) was pre-incubated with EcCysE (1.5 to 6 µM) for 25 min prior to the addition of CdiA-CT (0.5 µM). After further incubation for 25 min, reactions were initiated by addition of total *E. coli* RNA to a final concentration of 2 µg µL^−1^. Reactions were quenched with SDS-formamide gel-loading buffer after 10 min. CysK (0.1 to 10 µM) enzymes from other bacteria species were assayed in the same manner, except that CdiA-CT was used at 1 µM final concentration and the reactions were quenched after 1 h. All reactions were run on 8 M urea, Tris-borate-EDTA polyacrylamide gels, and RNA visualized by ethidium bromide staining.

## Electronic supplementary material

Supplemental Material
