# Supplementary material for: Activation of an anti-bacterial toxin by the biosynthetic enzyme CysK: mechanism of binding, interaction specificity and competition with cysteine synthase
Source: Sci Rep. 2017 Aug 18;7:8817. doi: 10.1038/s41598-017-09022-6 (PMC5562914; doi:10.1038/s41598-017-09022-6)
Supplement: Supplementary file 1 — Supplemental Material [file 41598_2017_9022_MOESM1_ESM.doc]

**Supplementary Information**

**Activation of an anti-bacterial toxin by the biosynthetic enzyme CysK: mechanism of binding, interaction specificity and competition with cysteine synthase**

Roberto Benoni^1#a^, Christina M. Beck^2#b^, Fernando Garza-Sánchez^2^, Stefano Bettati^1,3^, Andrea Mozzarelli^4,3^, Christopher S. Hayes^2,5^* and Barbara Campanini^4^*

^1^Dipartimento di Medicina e Chirurgia, Università di Parma, Parma, Italy

^2^Department of Molecular, Cellular and Developmental Biology, University of California, Santa Barbara, Santa Barbara, CA, USA

^3^Istituto Nazionale Biostrutture e Biosistemi, Rome, Italy

^4^Dipartimento di Scienze degli Alimenti e del Farmaco, Università di Parma, Parma, Italy

^5^Biomolecular Science and Engineering Program, University of California, Santa Barbara, Santa Barbara, CA, USA

^#a^ current address: Institute of Organic Chemistry and Biochemistry of the Czech Academy of Sciences, Praha, Czech Republic

^#b^ current address: Icahn School of Medicine at Mount Sinai, New York, NY, USA

*corresponding authors: chayes@lifesci.ucsb.edu (CSH) and barbara.campanini@unipr.it (BC)

**Table S1. Oligonucleotides**

| **Identifier** | **Description** | **Sequence*^a^*** |
| --- | --- | --- |
| CH2094 | Bsub-cysK-Spe | 5´ – GCT TAC TAG TAT CGA ATT GGT ACA GCG GCG |
| CH2095 | 3937-cysK-Spe | 5´ – GGA ACT AGT CTG CTG CAG TTC CTG TTC GG |
| CH2096 | Bsub-cysK-Nco | 5´ – GTC GAC CAT GGT ACG TGT AGC AAA CTC C |
| CH2099 | ECL-cysK-Spe | 5´ – GCT GAC TAG TCT GTT GCA GTT CTT TCT CGG |
| CH2101 | ECL-cysK-Nco | 5´ – GGC CAT GGG TAA GAT TTA TGA AGA CAA C |
| CH2102 | 3937-cysK-Nco | 5´ – CGG GCC ATG GGT AAG ATC TAC GAA GAC |
| CH2797 | cysK-Xho-rev | 5´ – TTT CTC GAG GGC ATT ACT GTT GCA ATT C |
| CH3345 | Nlact-cysK-Nco | 5´ – AGA CCA TGG AAA TTG CAA ACA GCA TCA CC |
| CH3346 | Nlact-cysK-Xho | 5´ – AAA CTC GAG CGC CAA ATC GGC AAA CAG GGG CG |
| CH3466 | Hin-cysK-Nco | 5´ – ATA CCA TGG CAA TTT ATG CAG AC |
| CH3467 | Hin-cysK-Spe | 5´ – TTT ACT AGT TCC CTC AAT CCC TTC AAA C |
| CH3865 | cysK-Kpn-for | 5´ – TTT GGT ACC AGT AAG ATT TTT GAA GAT AAC TCG |
| CH3642 | cysE-Kpn-native-for | 5´ – TTT GGT ACC ATG TCG TGT GAA GAA CTG G |
| CH4125 | cysE-∆11-Xho-rev | 5´ – ATG CTC GAG TTT AAC CGT TGA AAT GCT GGT CC |
| CDI235 | cysE-Xho-rev | 5´ – GAT CTC GAG ACA TTA GAT CCC ATC CCC ATA CTC |

*^a^*Restriction endonuclease recognition sequences are underlined

**Supplemental figure legends**

**Figure S1. Comparison of pre-steady state kinetics of cysteine synthase and EcCysK:CdiA-CT complex formation.** Dependence of k_obs_ on the concentration of EcCysK for the formation of EcCysK:EcCysE (closed triangles) and EcCysK:CdiA-CT (closed circles) complexes. Experiments were conducted with 270 nM CdiA-CT or 400 nM EcCysE in buffer A at 20 °C. Solid line represents the fitting to a linear equation with a slope 4.10 ± 0.1 µM^-1^·s^-1^ and 0.017 ± 0.007 µM^-1^·s^-1^ for CS and EcCysK:CdiA-CT complex formation, respectively.

**Figure S2. Densitometric analysis of native PAGE.** The native PAGE gel in Fig. 4C was analyzed with Image Lab software as described in Materials. The band % is calculated as the volume of each band compared to the sum of all band volumes in each lane. The analysis is shown in the right panels for two representative lanes. The relative band percent volume is also presented as a histogram in the lower left panel.

**Figure S3. CysK protein alignment.** The sequences of EcCysK, DdCysK, ECLCysK, BsCysK, HiCysK and NlCysK were aligned using ClustalW with Blosum matrix set at default parameters. Similarity scores were calculated by the ESPript program[^1^](#_ENREF_1) using the Blosum62 matrix set at global score of 0.2. Residues comprising the first active-site shell are indicated by red stars below the alignment. Secondary structure elements depicted above the alignment are derived from the crystal structure of the EcCysK:CdiA-CT binary complex (PDB code: 5J43). Residues on blue background are involved in direct interactions with CdiA-CT as reported in[^2^](#_ENREF_2).

**Figure S4. Analysis of proteins used in this study. A**) SDS-PAGE analysis of protein preparations used in this study. **B**) Secondary structure of refolded CdiA-CT and CdiI-His_6_. Circular dichroism spectra were acquired on samples containing 0.23 mg mL^-1^ protein in 20 mM sodium phosphate (pH 7.0). Dashed grey lines represent the result of spectra deconvolution using Dichroweb analysis Webserver[^3^](#_ENREF_3).

FIGURE S1

**
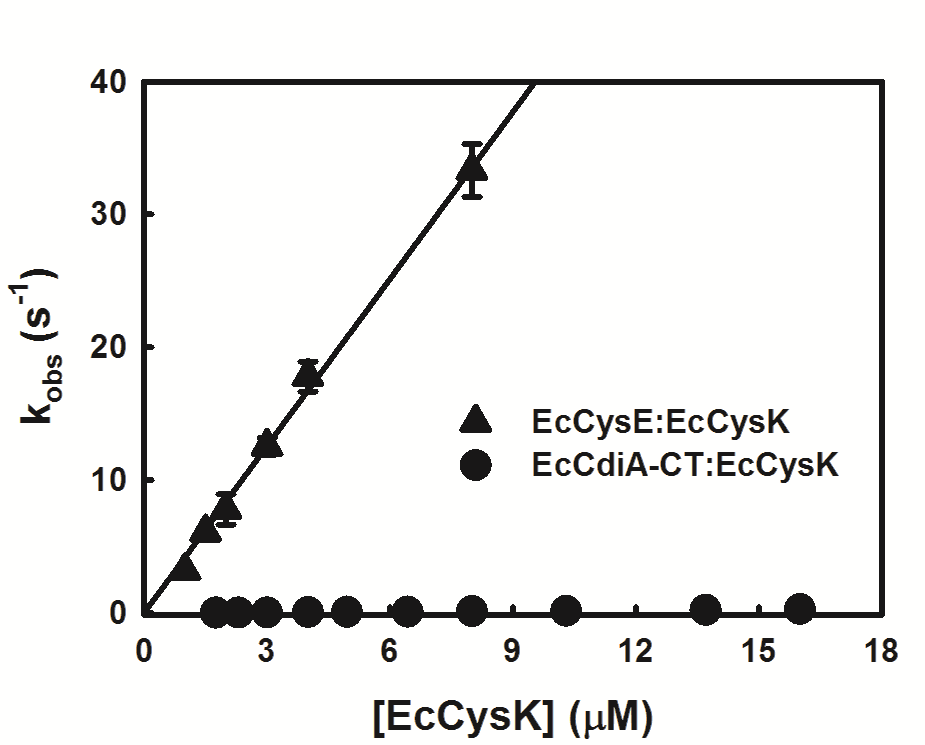
**

FIGURE S2

**
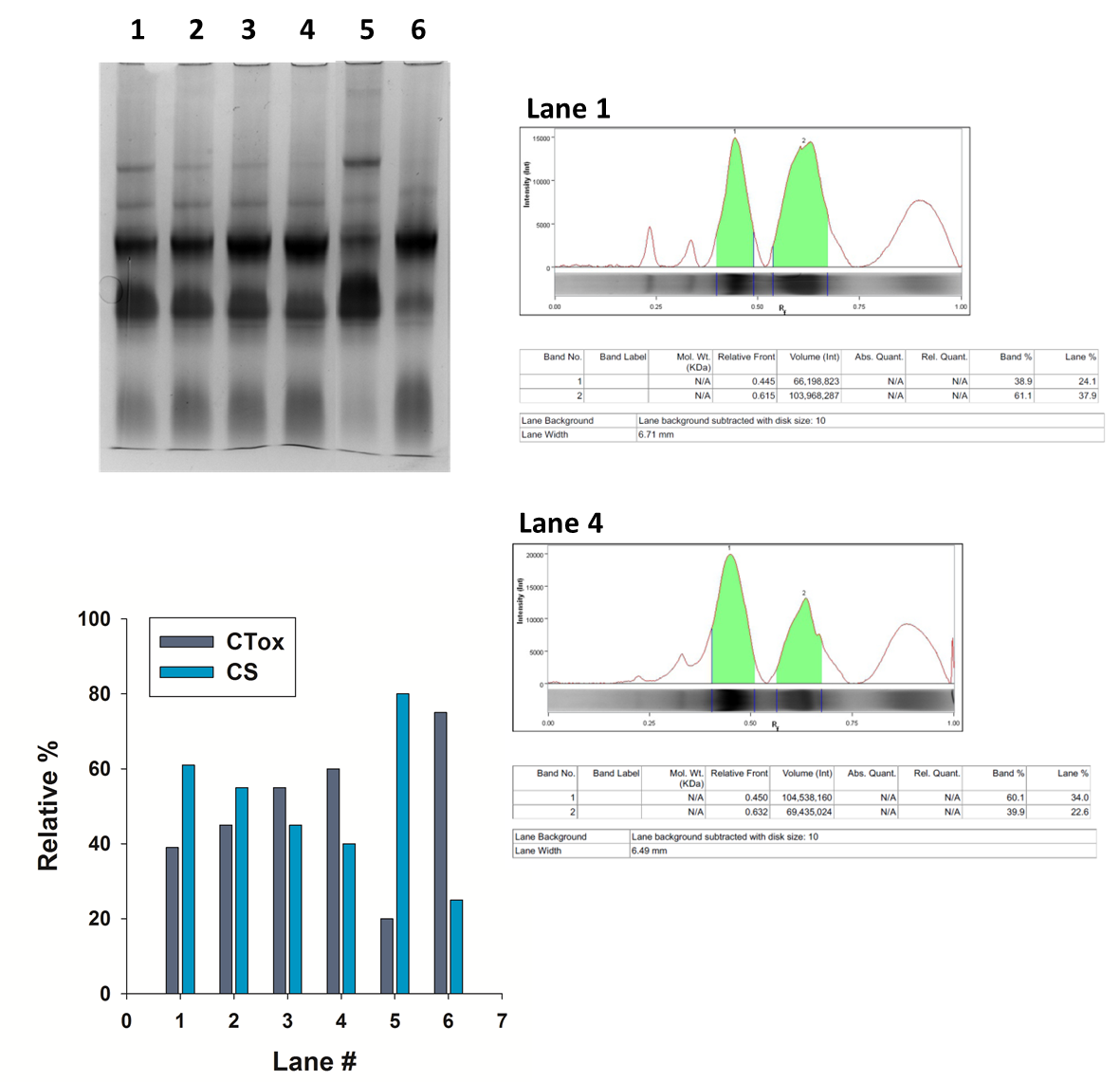
**

FIGURE S3


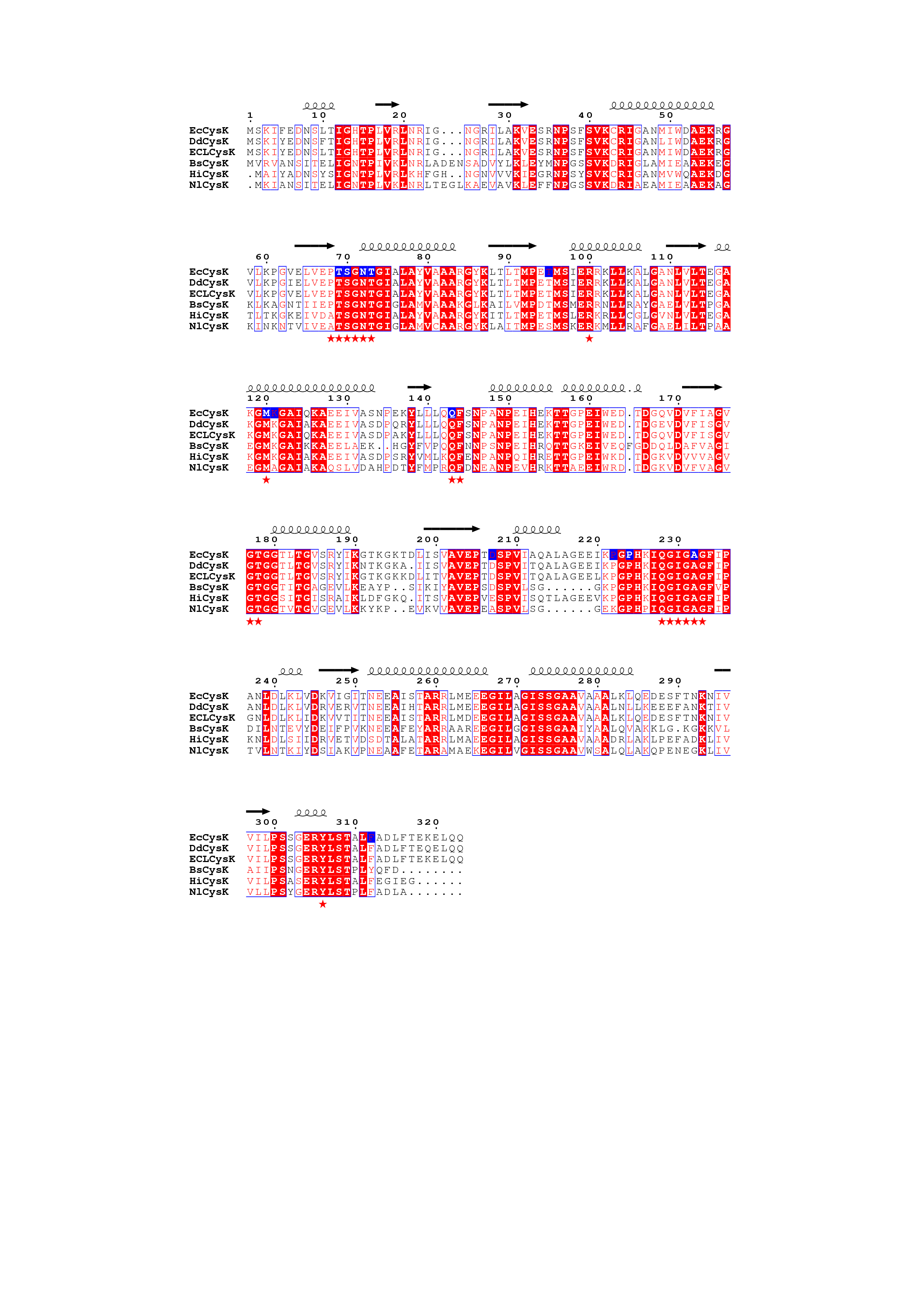


FIGURE S4

**
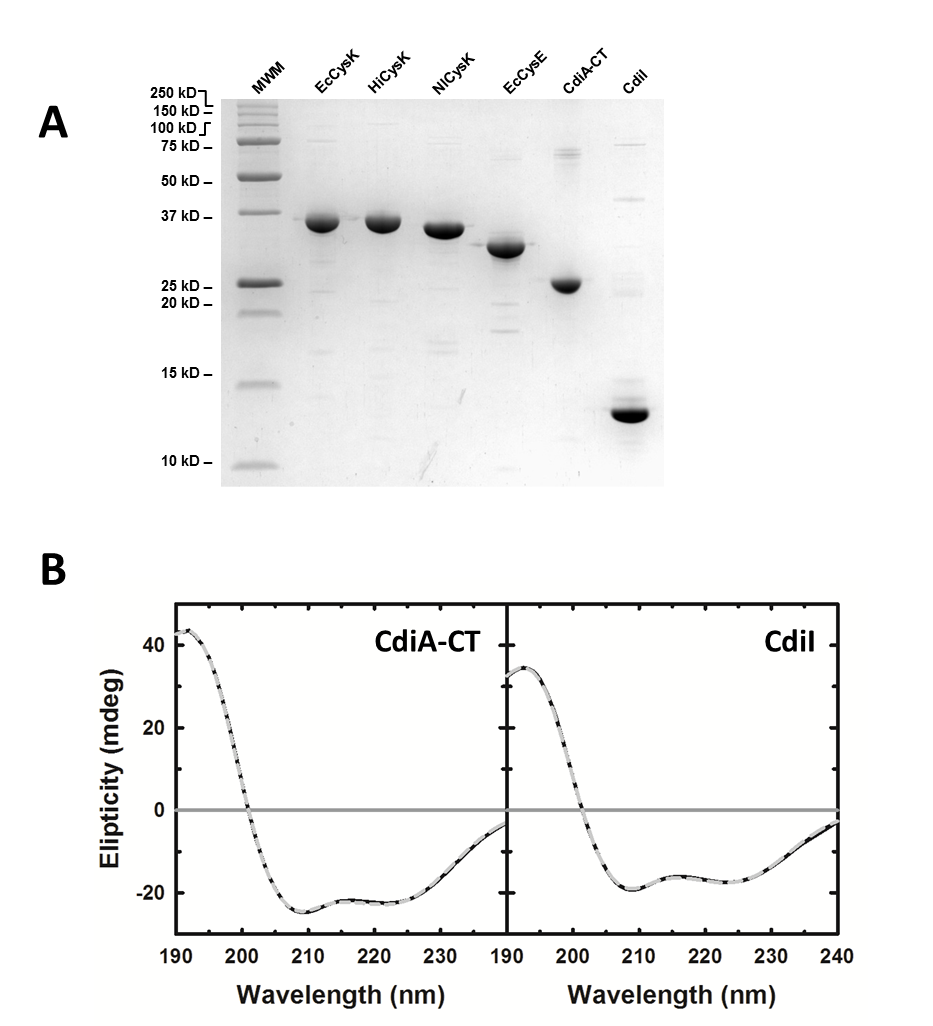
**

1. Gouet, P., Courcelle, E., Stuart, D.I. & Metoz, F. ESPript: analysis of multiple sequence alignments in PostScript. *Bioinformatics* **15**, 305-8 (1999).

2. Johnson, P.M. et al. Unraveling the essential role of CysK in CDI toxin activation. *Proc Natl Acad Sci U S A* **113**, 9792-7 (2016).

3. Whitmore, L. & Wallace, B.A. Protein secondary structure analyses from circular dichroism spectroscopy: methods and reference databases. *Biopolymers* **89**, 392-400 (2008).
